# Progress in Bacteriology

**Published:** 1902-01-18

**Authors:** 


### Progress in Bacteriology.

**The Tubercle Bacillus.**—The exact temperature to which milk must be raised in order to destroy tubercle bacilli which may be present has always been a disputed point. Bang stated that 85° C. was sufficient, Foster 70° C. for 10 minutes, de Man 80° C. for four minutes, whilst Galtier maintained that the tubercle bacillus could in this medium resist a temperature of 85° C. for six minutes. Barthel and Strenström [1] find that these differences depend upon differences of reaction of the milk. The milk from cows with advanced tuberculosis of the udder is more alkaline than that of healthy cows, or those only lightly infected. Tubercle bacilli, like other micro-organisms, have greater powers of resistance when growing in an alkaline medium, and consequently in a heavily-infected alkaline sample a temperature of 80° maintained for 10 minutes may be insufficient to kill the tubercle bacilli present. In a commercial pasteuriser Farrington and Russell [4] found milk heavily laden with tubercle bacilli and kept for five minutes at a temperature of 140°F. was still infective, but was innocuous if the time were prolonged to 10 minutes. For safety a full 20 minutes should be allowed, the vessel being closed to prevent the formation of a skin upon the surface of the milk. The differences between human and bovine tubercle bacilli may best be studied from cultures grown on dogs' serum containing 5 per cent. of glycerine. Ravenel [2] finds that the bovine bacillus thus grown is short, thick, straight, and shows no beading. Bovine tubercle grows far less readily than the human variety on glycerine agar, and can seldom be transplanted on to this medium from blood serum tubes. Bovine tubercle is much more virulent than human to all animals except swine, which react equally to both, and the two varieties when isolated again from the animals experimented upon show distinct morphological differences. Of four calves experimentally infected with human tuberculous sputum, three developed extensive tuberculous lesions. The attempts to infect calves by feeding experiments failed. Ravenel believes that man may be infected by bovine tubercle, and records eight undoubted cases of local infection of this nature, in two of which the infection became general and death ensued. The serum diagnosis of tuberculosis is still only in the trial stage. The matter has recently been reviewed exhaustively by Romberg,[3] and his conclusions are generally unfavourable to this procedure. On Behring's suggestion he used emulsions of dead bacilli.

**Acute Rheumatism.**—Singer [5] found streptococci in the tissues of five cases of fatal articular rheumatism, and staphylococci in a sixth case in which suppuration of the elbow joint supervened. Mayer [6] isolated a diplococcus growing in chains from cases of acute follicular tonsillitis, and cultures injected into animals excited a sterile seropurulent exudation into the joints, and in one-fifth of the cases ulcerative endocarditis. A similar organism was found in three out of four cases by Menzer.[7] There would appear to be an increasing volume of evidence in favour of the belief that acute rheumatism is a specific infective disorder due to a diplococcoid infection.

**Enteric Fever.**—Remlinger first demonstrated the possibility of infecting animals with typhoid fever by a series of experiments on rats. Ainley [8] has now shown the same thing to be true of rabbits, and further demonstrated that anti-typhoid serum has both protective and curative action, whilst tissue extracts obtained from the organs of animals immunised against the B. typhosus conferred no such protection. Savage [9] has examined the causes which lead to pseudo-clumping of the typhoid bacillus, a condition causing much inconvenience and possibly error in performing Widal's test. He eliminates many supposed causes, such as alkalinity of the medium, age of the culture, prolonged incubation, etc., and finds that there are two main factors, viz., susceptibility of the race of B. typhosus, and the use of broth subcultures. The altered constituents of the broth which cause this pseudo-clamping are not known, but agar cultures never give such results.

**Membranous Tonsillitis.**—Sobel [10] reports twelve cases of ulcero-membranous angina due to the fusiform bacillus (Vincent). This organism associated with spirilla may excite also certain forms of stomatitis, the ulcers being found on the tongue, cheeks, or gums. For purposes of diagnosis films are more to be relied on than cultures, and the best stain for films is carbol-fuchsin. Whilst it is usual to find fusiform bacilli and spirilla together, occasionally only bacilli may be present. The fusiform bacillus is twice as long as the diphtheria bacillus and tapers at the ends. It is motile, does not form spores and is slowly decolorised by Gram. In taking a specimen the swab should be rubbed deeply into the ulcer. The fusiform bacillus does not grow on ordinary media but has been obtained in impure culture in peptone-bouillon and in organic fluids from the human body. Cultures give rise to a fœtid odour, and when inoculated set up suppurative lesions. Its thermal death point is 60° C. The author believes that the bacillus is only an involution form of the spirillum, or vice-versa, and that this organism is the direct and specific exciting cause of this condition of ulcero-membranous angina. Bissell[11] described three organisms other than the diphtheria bacillus capable of exciting membranous tonsillitis, namely, streptococcus pyogenes, oïdium-albicans and the micrococcus of sputum septicæmia. Although clinically the oïdium infection most closely simulated diphtheria owing to the wide distribution of the membrane, yet it was never fatal as were the other two infections. The spirochœta bacillus described by Solomon,[12] is probably identical with the spirillum of Vincent. Solomon describes four cases of membranous tonsillitis in which B. spirochœta was the predominating organism. It is never found with the Klebs Löffler bacillus, but is frequently met with in the shallow ulceration of secondary syphilis, and in cases of stomatitis. The writer mentions that it is sometimes associated with the fusiform bacillus, but failed to obtain cultures on any media.

**Fœtid and Gangrenous Suppuration.**—In many cases of fœtid and gangrenous suppuration if cultures be made upon the ordinary media, only a simple coccal or bacterial infection is revealed, but if from the same cases direct coverglass films be prepared and stained a large variety of cocci, bacilli, vibrios, and spirochetes may be seen, the new varieties revealed being unable to grow in the media provided for them. Rist[13] has studied these pleomorphic infections with the special purpose of developing any anærobic organisms present, and has used for this purpose deep tubes of solidified glucose gelatine, He concludes that most of the cases of fœtid otorrhœa and mastoiditis with secondary lung infection are due to anærobic bacteria, though a few may have streptococcal or pneumococcal origin. In 19 out of 21 cases of appendicitis the pus contained anærobic germs, and these were also common in periurethral and periuterine suppurations. An interesting point was that certain strepto and ostaphylococci are anærobic. Amongst named forms are B. racemosus and B. fragilis (common in appendicitis and pulmonary gangrene), staphylococcus parvulus, micrococcus fœtidus, and B. bifidus. Though generally associated with ærobic organisms these anærobes may exist and excite suppuration alone, and are invariably present in fœtid suppuration and active gangrene. Their presence always warrants a grave prognosis, and should lead to treatment with oxidising antiseptics such as permanganate of potash, or peroxide of hydrogen.

[1] Centrab. f. Bakt, Oct. 8, 1901. [2] Univ. of Penn. Med. Bull., Sept., 1901. [3] Deut. Med. Woch., May 2, 1901. [4] Public Health, Oct., 1901, p. 27. [5] Wien. Klin. Woch., May 16, 1901. [6] Deut. Med. Woch., Feb. 7, 1901. [7] Ibid., Feb. 14, 1901. [8] Jour. of Path. and Bact., Nov., 1901. [9] Ibid. [10] New York Med. Jour., Dec., 1901. [11] Buff. Med. Jour., Dec., 1901. [12] Deut. Med. Woch., Aug. 22, 1901. [13] Brit. Med. Jour., Oct. 12, 1901.

